# The low prevalence effect in fingerprint comparison amongst forensic science trainees and novices

**DOI:** 10.1371/journal.pone.0272338

**Published:** 2022-08-11

**Authors:** Bethany Growns, James D. Dunn, Rebecca K. Helm, Alice Towler, Jeff Kukucka

**Affiliations:** 1 College of Social Sciences and International Studies, University of Exeter, Exeter, United Kingdom; 2 School of Psychology, University of New South Wales, Sydney, Australia; 3 Department of Psychology, Towson University, Towson, MD, United States of America; Universiti Teknologi Malaysia - Main Campus Skudai: Universiti Teknologi Malaysia, MALAYSIA

## Abstract

The *low prevalence effect* is a phenomenon whereby target prevalence affects performance in visual search (e.g., baggage screening) and comparison (e.g., fingerprint examination) tasks, such that people more often fail to detect infrequent target stimuli. For example, when exposed to higher base-rates of ‘matching’ (i.e., from the same person) than ‘non-matching’ (i.e., from different people) fingerprint pairs, people more often misjudge ‘non-matching’ pairs as ‘matches’–an error that can falsely implicate an innocent person for a crime they did not commit. In this paper, we investigated whether forensic science training may mitigate the low prevalence effect in fingerprint comparison. Forensic science trainees (*n* = 111) and untrained novices (*n* = 114) judged 100 fingerprint pairs as ‘matches’ or ‘non-matches’ where the matching pair occurrence was either high (90%) or equal (50%). Some participants were also asked to use a novel feature-comparison strategy as a potential attenuation technique for the low prevalence effect. Regardless of strategy, both trainees and novices were susceptible to the effect, such that they more often misjudged non-matching pairs as matches when non-matches were rare. These results support the robust nature of the low prevalence effect in visual comparison and have important applied implications for forensic decision-making in the criminal justice system.

## Introduction

Forensic science examiners often complete visual comparison tasks where they compare items of evidence (e.g., fingerprints, bullets, handwriting samples) and opine as to whether they originated from the same source or different sources [1]. For example, fingerprint examiners compare a suspect’s fingerprint against a latent fingerprint found at a crime scene and decide whether they belong to the same person (i.e., ‘match,’ implying guilt) or different people (i.e., ‘non-match,’ implying innocence). These judgments are influential in criminal investigations [2, 3] but are also perceptually and cognitively complex [4], especially when the evidence is of relatively poor quality (e.g., incomplete or smudged latent fingerprints). Despite its difficulty, professional fingerprint examiners outperform novices in experimental settings [5, 6]. However, even professional examiners still make errors that can result in costly miscarriages of justice [7]. It is therefore vital to investigate ways to minimise errors in forensic science judgments.

Prior work has identified numerous factors that can produce errors in forensic decision-making, such as the context in which the evidence is presented [8–10] or personal factors like fatigue and stress [11, 12]. Another potential source of error is the relative base-rates of ‘matching’ and ‘non-matching’ samples–something that has the potential to create a *low prevalence effect*. This is a well-documented phenomenon in cognitive science research where people more often ‘miss’ (i.e., fail to detect) rare targets in visual search or comparison tasks [13–17]. For example, airport security officers can fail to detect weapons in baggage when weapons appear infrequently [18], or people failing to detect cancer in mammograms when cancer is rare [19].

Research has demonstrated that the low prevalence effect is also pervasive in visual comparison tasks. Growns and Kukucka [15] asked untrained novices to compare 100 fingerprint pairs and decide if they were from the same person or different people, whilst manipulating whether ‘matching’ pairs were relatively rare (i.e., 10% of trials), common (i.e., 90% of trials) or were equally likely to occur as to ‘non-matching’ pairs (i.e., 50% of trials). When ‘match’ prevalence was high, novices become more likely to misjudge non-matching pairs as matches compared to equal, and vice versa when ‘non-match’ prevalence was high. These effects were not driven by changes in sensitivity (i.e., sensitivity to the presence of a target stimulus), but rather by criterion shifts (i.e., response biases) in novices’ tendency to respond ‘match’ on any given trial when match trials were more common, and vice versa when non-match trials were more common. These results mirror effects also seen in face comparison [16, 17, 20, 21]; although see [22] for an exception).

The low prevalence effect has proven to be remarkably robust; prior attempts to correct this bias–such as allowing participants to correct their answers [23, 24], imposing slower responding [25, 26], and explicit warnings [27]–have largely failed. Moreover, prior studies in non-forensic domains have found that novices and professionals are equally susceptible to the low prevalence effect, including TSA baggage screeners [18], security professionals [21], and doctors [28]–which suggests that standard training and experience fail to protect against it.

In real-world fingerprint casework, it is thought that matching and non-matching fingerprint pairs do not occur equally [29, 30] Although it is important to note the true base-rates in the criminal justice system cannot be known, examiners likely view matching pairs much more often than non-matching pairs [29, 30]. This could increase false-positive errors where innocent suspects are falsely accused of a crime. However, no research has investigated whether forensic science education or training has the potential to inoculate them against such a bias. The primary aim of the current study is to address this question by comparing susceptibly to the low prevalence effect in fingerprint comparison between untrained novices and forensic science students. This would be evidenced by an elevated error rate on non-match trials when non-matches are rare, and a corresponding smaller criterion shift.

As a secondary aim, we also test the use of a feature-comparison strategy as a novel technique for reducing the low prevalence effect in fingerprint comparison. The feature-comparison strategy requires individuals to slowly and deliberately break down and compare the discrete features of two visual stimuli [31, 32] rather than viewing them holistically [33, 34]. This strategy has been shown to improve facial comparison accuracy: people who rate the similarity of facial features have higher accuracy than those making no ratings or control ‘image quality’ ratings. The feature-comparison strategy is particularly effective at improving performance on non-match trials [32]–the precise trial-type where the low prevalence effect is thought to occur in casework [29]. Given visual comparison is a generalisable ability with comparable underlying cognitive mechanisms ([33], see [34] for review), it is possible that feature-comparison may also be a technique that could: a) improve fingerprint comparison performance; and b) reduce the low prevalence effect by decreasing errors on non-match trials are rare. If this strategy benefits performance, it could easily be adapted and incorporated into existing training programs for forensic examiners. As a secondary aim, we will also test whether the use of a feature-comparison strategy reduces the low prevalence effect in fingerprint comparison, and whether the effect of using this strategy differs between trainees and novices.

## Method

### Ethics statement

This study was approved by the University of Exeter College of Social Sciences and International Studies Ethics Committee. All participants confirmed that they had read the study information sheet and provided informed written consent online.

### Design

The current study used a 2 (trainee vs. novice) x 2 (match prevalence: high [90%; i.e., non-match prevalence is low, or 10% of trials] vs. equal [50%]) x 2 (strategy: feature-comparison vs. control) between-subjects design. Each trainee or novice participant was randomly assigned to one of the four prevalence x strategy cells. The study pre-registration https://osf.io/tes2k, and data and analysis scripts can be found at https://osf.io/g2zdm/.

### Participants

We recruited 224 participants online, based on an *a priori* power analysis for detecting a medium effect with 80% power in our 2 x 2 x 2 between-subjects design (*n* = 196), including 10% to account for attrition (*n* = 20; desired *N* = 216). Note that while we pre-registered that trainees would be defined as students who indicated they aspired to fingerprint examination as a career, only a slight minority of our final sample actually met this criterion (*n =* 53, 46%). As per our pre-registered analysis plan, we thus expanded our definition of ‘trainees’ to all participants who indicated that they had training or education in forensic science for all analyses reported in-text (*n* = 116). We report our pre-registered analyses of trainees who aspired to a career as a fingerprint examiner (our initial pre-registered definition of ‘trainee’) in S1 Text on OSF; and several additional exploratory analyses: 1) comparing novices and only trainees who reported having some training in fingerprint examination specifically (*n* = 58, 50%); 2) comparing trainees with or without study or training in fingerprint examination; and 3) controlling for gender due to the gender imbalance in the trainee sample (see below). Importantly, the results of these analyses did not differ from those reported in-text (see S1 Text on OSF).

Trainees were recruited via a snowball-sampling method with emails sent to forensic science training and education programs in the UK, US, and Europe (*n* = 118). One trainee was excluded for reporting having no training or education in forensic science, and one trainee was excluded based on our pre-registered criterion of responding incorrectly to at least two of three attention-check questions (e.g., *‘Please select the [same/different] option below’* with artificial images). Novices were recruited from Prolific Academic (*n* = 109), and all reported having no training or study in forensic science. In exchange for approximately 60 minutes of participation, all participants received £10 (or the equivalent in USD or Euros) via either Prolific Academic credit (novices) or an electronic Amazon voucher (forensic trainees). Participants recruited from Prolific were also required to live in the United Kingdom, have normal or corrected-to-normal vision, and have a Prolific approval rating of at least 90% (i.e., at least 90% of the studies each individual had previously participated in were approved by the experimenter). Although we could not apply these same selection criteria to our trainee sample due to the different recruitment method, the inclusion criterion for trainees was that they reported being a current student working toward a degree or certification in forensic science All participants were required to complete the study on a computer or tablet (i.e., not a cellular device).

Trainees in the final sample (*n* = 116) were on average 21.54 years old (*SD* = 4.11, *range* = 18–48), and most (83.62%) self-identified as female (15.52% male; 0.90% gender diverse). The average trainee reported having 20.07 months of experience (*SD* = 12.09, *range =* 2–45) in their current forensic science degree or certification program. As noted above, 50% of trainees (*n* = 58) reported having training in fingerprint examination specifically, and they averaged 4.83 months of training in that discipline (*SD* = 6.39, *range* = 1–24). Novices in the final sample (*n* = 108) were on average 35.14 years old (*SD* = 11.67, *range* = 18–63) and a half of participants self-identified as male (48.15% female; 1.85% gender diverse).

### Materials and procedure

The procedure and materials were identical for trainees and novices. All participants completed the experiment via the online survey platform Qualtrics. After being randomly assigned to a condition, all participants first received instructions adapted from [15] that explained the upcoming fingerprint comparison task, which read as follows:

*“Fingerprint examiners compare fingerprints found at crime scenes against suspects’ fingerprints to determine whether they match. If a crime scene fingerprint and a suspect fingerprint are similar, this would suggest that they are the same person. Conversely, if they are dissimilar, this would suggest that they are different people. In this study, you will perform the role of a fingerprint examiner by comparing two fingerprints and deciding if they are from the same person or two different people. You will be asked to select from one of two options when making this decision: ’same person’ or ’different people’. Please complete each comparison as quickly and accurately as you can.”*

Next, all participants completed two practice trials (one match and one non-match) adapted from [15] using computer-generated fingerprints and received corrective feedback after each trial. These practice trials were included as a way for participants to familiarise themselves with the survey website and task.

#### Strategy manipulation

**Fig 1:**
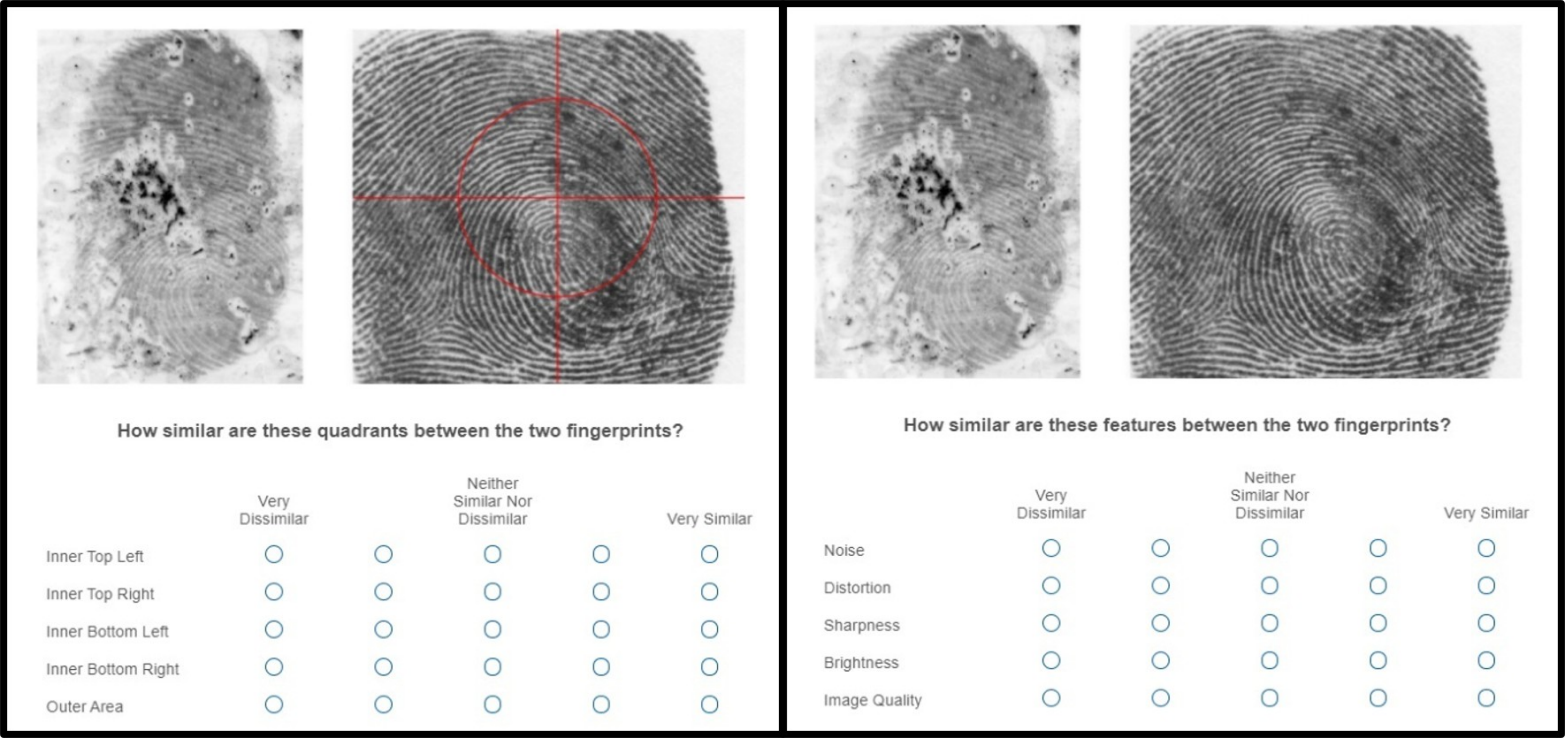
An example trial from the feature-comparison (left) and control (right) conditions. The red grid illustrated in the left panel was overlaid to highlight each of the five areas participants in the feature-comparison condition were asked to rate (i.e., inner upper left, inner upper right, inner lower left, inner lower right, and outer area).

Then, all participants received additional instructions explaining the ratings that they would be asked to provide for each fingerprint pair. Participants in the *feature-comparison* strategy condition were told that they would be asked to rate the similarity of each of five areas (i.e., inner upper left, inner upper right, inner lower left, inner lower right, and outer area; instructions adapted from [32] between the two fingerprints, each on a Likert scale ranging from 1 (*very dissimilar*) to 5 (*very similar*). As shown in the left panel of Fig 1, participants in the feature-comparison condition viewed each exemplar (i.e., the rolled, clear print) fingerprint (right) with a red grid overlaid to distinguish these areas, and they were instructed to do their best to locate the corresponding regions in the latent print (left) for comparison purposes, as the two prints may differ by angle or orientation.

Participants in the control strategy condition were told that they would be asked to rate the similarity of five image quality aspects (i.e., noise, distortion, sharpness, brightness, and image quality) between the two fingerprints (see right panel of Fig 1). These instructions also provided definitions of each of these terms (adapted from [31]):

- *Noise*: refers to random dot/pixel level variations in the images as in how “noisy” each image is. You can think of this as the visual “noise” than is seen on a television without a signal.
- *Distortion*: refers to differences between the shapes of the two fingerprints.
- *Sharpness*: refers to differences in how clear or blurry each fingerprint is.
- *Brightness*: refers to differences in how bright or dark each fingerprint is.
- *Image quality*: refers to how well the image captures the overall fingerprint.

We requested these ratings so that participants in the control strategy condition would complete a similar (but non-informative) task for each fingerprint pair as those in the feature-comparison condition.

#### Fingerprint stimuli

**Fig 2:**
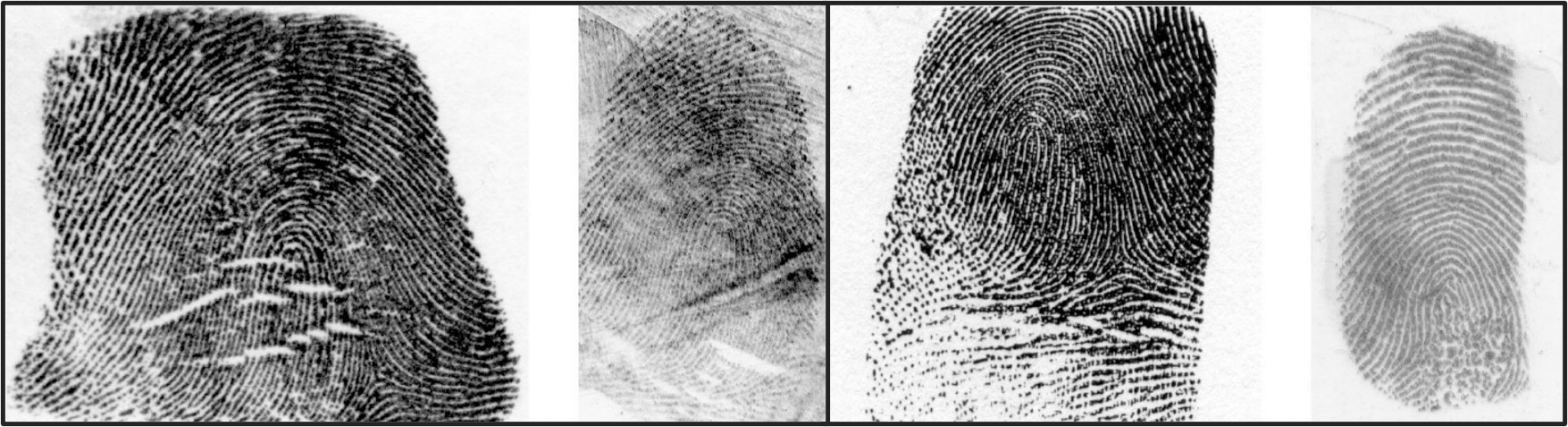
Example match (left panel) and non-match (right panel) trials between exemplar (left image in each example) and latent (right image in each example) fingerprints.

Next, participants viewed and judged the same 100 fingerprint pairs used in [15]. Each trial consisted of one exemplar fingerprint (left) and one latent fingerprint (right) shown side-by-side (see Fig 2 for examples of matching and non-matching pairs). For each, participants first provided the similarity ratings relevant to their strategy condition, and then they indicated their opinion as to whether the two fingerprints belonged to the ‘same person’ or ‘different people’ by clicking one of two buttons at the bottom of the screen. After providing each binary judgement, participants received corrective feedback (i.e., correct or incorrect) before advancing to the next trial.

#### Prevalence manipulation

Participants were randomly assigned to complete either 90 match trials and 10 non-match trials (*high match prevalence* condition) or 50 match trials and 50 non-match trials (*equal match prevalence* condition). Within each prevalence condition, each participant completed all 100 trials in the same pseudo-randomised order to minimise error variance [35] where one trial order was randomly generated when coding the experiment; in the equal match prevalence condition, the first 20 trials included exactly 10 match trials and 10 non-match trials. In addition, Qualtrics recorded participants’ response latencies for each of the five ratings as well as the binary match/non-match judgement. After rating and judging all 100 pairs, participants provided demographic information and were debriefed.

### Dependent measures and analyses

We coded participants’ judgements according to a signal detection framework. For match trials, a correct judgment was coded as a ‘hit’ and an incorrect judgment was coded as a ‘miss’ (see Table 1). For non-match trials, a correct judgment was coded as a ‘correct rejection’ and an incorrect judgment was coded as a ‘false alarm’ (see Table 1). We also calculated signal-detection measures of sensitivity (d’) and bias (C). Higher d’ values indicate better sensitivity to the presence of a target stimulus (i.e., a higher ratio of hits to false alarms). Positive C values indicate an increased tendency to answer ‘non-match,’ whilst negative C values indicate an inclination to answer ‘match,’ irrespective of accuracy [36, 37].

**Table 1:** Signal detection framework of the correct decisions and errors that can be made in forensic feature comparison decisions.

		Ground Truth	
		Match	Non-Match
Decision	Match	*Hit*	*False alarm*
	Non-Match	*Miss*	*Correct rejection*

## Results

### Error rates analyses

To investigate the effects of training, prevalence, and strategy on fingerprint identification errors (false alarms and misses), we used the *lmer* [38] and *lmerTest* [39] R packages to create logistic mixed-effects models to predict each measure at the trial level from the interaction between prevalence (equal or high), strategy (feature-comparison or control), and group (novices or trainees). We included random effects for trial and participant, which allowed values to vary between stimuli and participants. See S1 Text on OSF for a table of all results of each analysis.

#### False alarms

**Fig 3:**
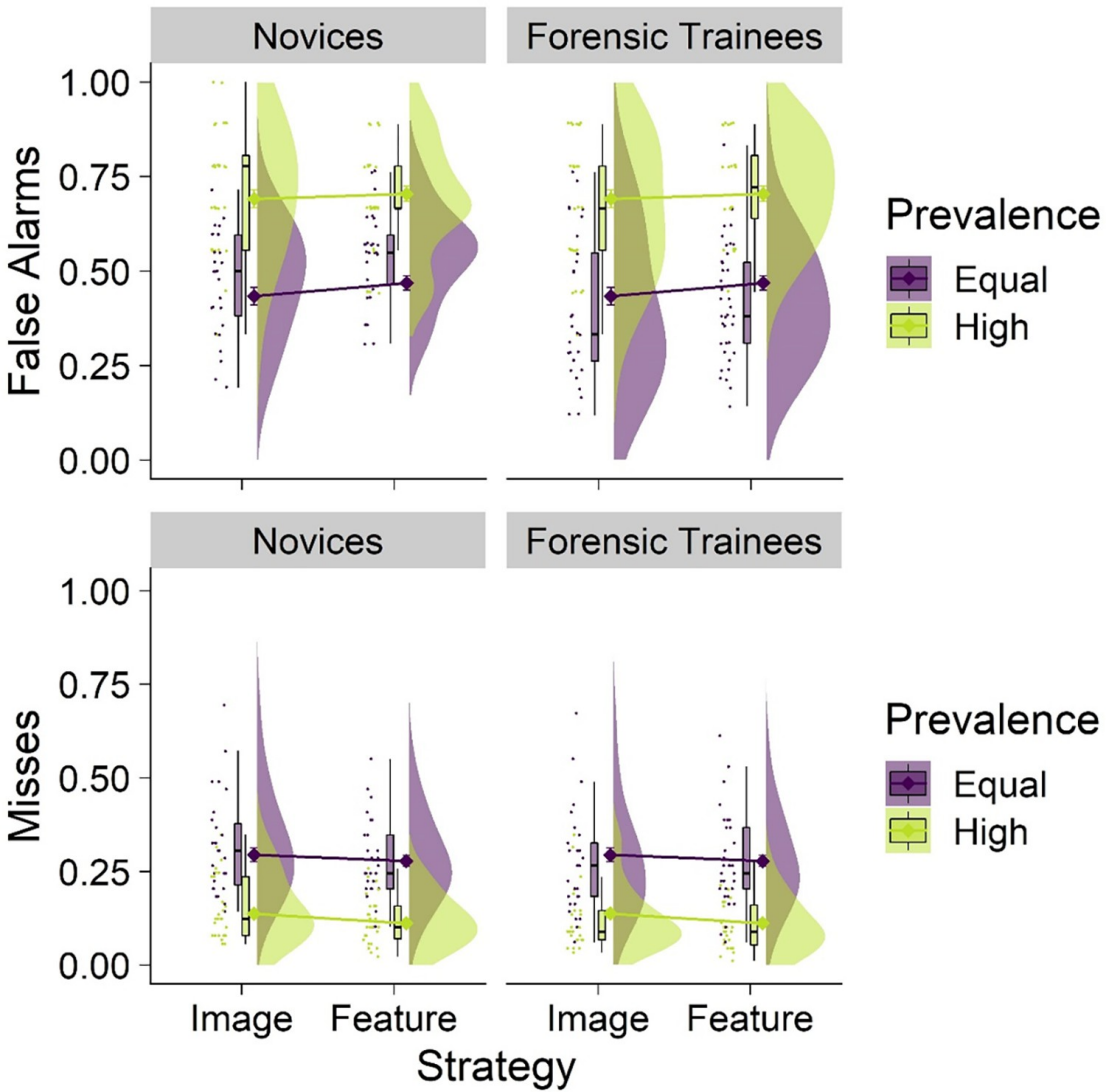
False alarms (top panel) and misses (bottom panel) by prevalence, strategy conditions, and group. Raincloud plots depict (left-to-right) raw jittered data points, box-and-whisker plots, means (represented by diamonds) with error bars representing ± 1 *SE*, and frequency distributions.

We replicated the low prevalence effect (see Fig 3), as the false alarm rate was significantly higher in the high prevalence condition (*M* = .70, *SD* = .46) than in the equal prevalence condition (*M* = .45, *SD* = .50; *b* = 1.71, *z* = 5.47, *p* < .001, 95% CI [1.10, 2.59]). The false alarm rates did not significantly differ between trainees and novices (*b* = -.42, *z* = 1.84, *p* = .065, 95% CI [-.86, .03]), nor did false alarm rates differ between strategy conditions (*b* = .38, *z* = 1.67, *p* = .096, 95% CI [-.07, .83]).

Importantly, there was no significant two-way interaction between prevalence and group on false alarms (*b* = -.01, *z* = .03, *p* = .980, 95% CI [-.78, .76]), indicating that both trainees and novices exhibited the low prevalence effect to the same degree. Likewise, there were no significant two-way interactions between prevalence and strategy (*b* = -.58, *z* = 1.48, *p* = .140, 95% CI [-1.36, .19]), or strategy and group (*b* = -.26, *z* = .84, *p* = .402, 95% CI [-.87, .35]), nor was there a significant three-way interaction (*b* = .88, *z* = 1.57, *p* = .117, 95% CI [-.22, 1.98]) for false alarm rates.

#### Misses

Consistent with the low prevalence effect, the miss rate was significantly lower in the high prevalence condition (*M* = .13, *SD* = .33) than in the equal prevalence condition (*M* = .29, *SD* = .45), *b* = -1.07, *z* = 5.38, *p* < .001, 95% CI [-1.45, -.68]). As with false alarm rates, miss rates did not differ between groups (*b* = -.31, *z* = 1.57, *p* = .117, 95% CI [-.70, .08]), or between strategy conditions (*b* = -.18, *z* = .90, *p* = .366, 95% CI [-.57, .21]).

There were no significant two-way interactions between prevalence and group (*b* = -.03, *z* = .12, *p* = .905, 95% CI [-.58, .52]), prevalence and strategy (*b* = -.12, *z* = .42, *p* = .677, 95% CI [-.67, .43]), or strategy and group (*b* = .25, *z* = .92, *p* = .360, 95% CI [-.28, .78]), nor was there a significant three-way interaction (*b* = -.09, *z* = .23, *p* = .816, 95% CI [-.87, .69]), on miss rates.

### Sensitivity and response bias analyses

We also used the *lm* function in the core stats package in R to conduct linear regression models to predict sensitivity and bias from the interaction between prevalence, strategy, and group.

#### Sensitivity

**Fig 4:**
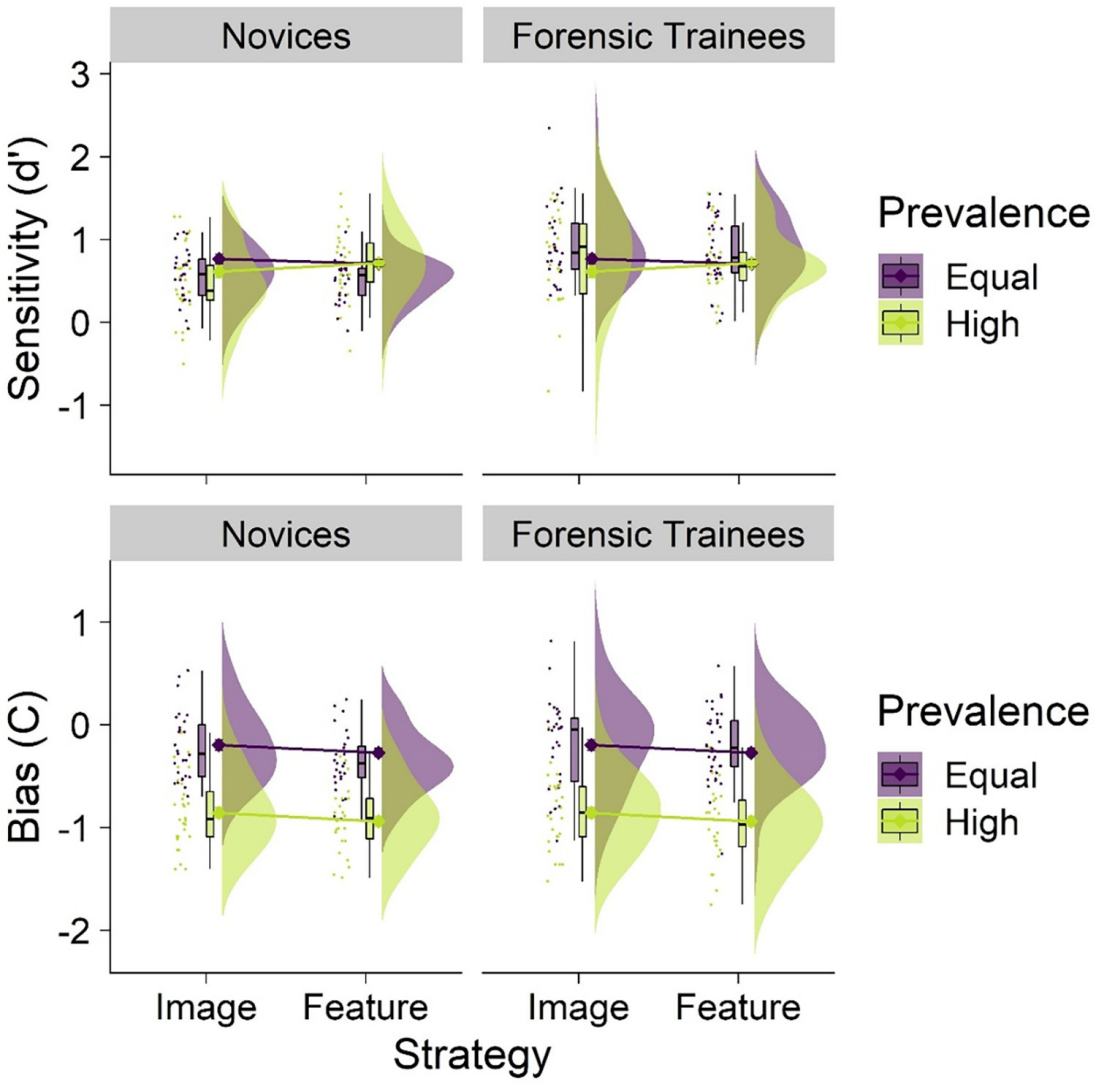
Sensitivity (top panel) and response bias (bottom panel) by prevalence, strategy conditions, and group. Raincloud plots depict (left-to-right) raw jittered data points, box-and-whisker plots, means (represented by diamonds) with error bars representing ± 1 *SE*, and frequency distributions.

Trainees had significantly higher sensitivity (*M* = .73, *SD* = .11) than novices (*M* = .70, *SD =* .12; *b* = .36, *t* = 3.28, *p* = .001, 95% CI [.15, .58]; see Fig 4). However, neither prevalence (*b* = -.11, *t* = .96, *p* = .341, 95% CI [-.33, .11]), nor strategy (*b* = -.07, *t* = .64, *p* = .525, 95% CI [-.30, .15]), affected sensitivity–suggesting that neither prevalence nor training improved fingerprint comparison performance.

There were no significant two-way interactions between prevalence and group (*b* = .07, *t* = .44, *p* = .659, 95% CI [-.38, .24]), prevalence and strategy (*b* = .31, *z* = 1.96, *p* = .051, 95% CI [-.02, .63]), or strategy and group (*b* = -.01, *t* = .07, *p* = .943, 95% CI [-.31, .29]), nor was there a significant three-way interaction (*b* = -.28, *t* = 1.24, *p* = .218, 95% CI [-.72, .16]) for sensitivity.

#### Response bias

The mean response bias value (C) was significantly lower in the high prevalence condition (*M* = -.90, *SD* = .35) than in the equal prevalence condition (*M* = -.24, *SD* = .36; *b* = -.64, *t* = 6.66, *p* < .001, 95% CI [-.83, -.45])–indicating a stronger propensity to judge fingerprint pairs as matches (irrespective of accuracy) in the high prevalence condition. Response bias did not significant differ between groups (*b* = .02, *t* = .24, *p* = .811, 95% CI [-.17, .21]), nor did strategy significantly affect response bias (*b* = -.15, *t* = 1.56, *p* = .121, 95% CI [-.34, .04]).

Notably, there was no two-way interaction between prevalence and group on response bias (*b* = -.03, *t* = .24, *p* = .809, 95% CI [-.30, .24]), indicating that high match prevalence created a similar response bias among both trainees and novices. Likewise, there were no significant two-way interactions between prevalence and strategy (*b* = .11, *z* = .77, *p* = .440, 95% CI [-.17, .38]), or strategy and group (*b* = .13, *t* = .99, *p* = .322, 95% CI [-.13, .39]), nor was there a significant three-way interaction (*b* = -.21, *t* = 1.10, *p* = .272, 95% CI [-.59, .17]) for response bias.

## Discussion

In this paper, we provide the first evidence of the low prevalence effect in forensic science trainees: both novices and forensic trainees were equally affected by the base-rates of ‘match’ and ‘non-match’ fingerprint pairs. Despite trainees’ overall performance advantage over novices, both groups made more false-alarms by misjudging non-matching pairs as matches when match prevalence was high (i.e., 90% of trials), compared to equal prevalence. Importantly, this is the same error that could result in the wrongful conviction of an innocent suspect in a crime [15, 40]. This effect was accompanied by fewer ‘misses’ where participants misjudged fewer matching pairs as non-matches when match prevalence was high, compared to equal prevalence. These effects were driven by a criterion shift where both trainees and novices over-compensated for the scarcity of the target, resulting in a stronger tendency to respond ‘match’ when match prevalence was high–irrespective of sensitivity [15, 16, 20].

These results add to evidence that forensic science decision-making can be impacted by task-irrelevant extraneous factors and cognitive bias (see [41] for review) and provide further evidence that standard forensic training does not inoculate against base rate-induced biases, as forensic trainees and novices were equally susceptible to the low prevalence effect. As the low prevalence effect was observed in both trainees and novices, these results also add to growing evidence that expertise or experience does not necessarily inoculate decision-makers against the low prevalence effect–a bias that has been identified amongst other professionals, including TSA baggage screeners [18], security professionals [21], and doctors [28].

We also examined whether engaging participants in a feature-comparison strategy would ameliorate the low prevalence effect in fingerprint comparison. This technique has been shown to increase face comparison accuracy (particularly on non-match trials where we have identified the low prevalence effect in fingerprint comparison; [30, 31]. However, we found no evidence that this strategy increased performance or decreased the low prevalence effect in fingerprint comparison. It is possible that the feature-comparison strategy is not effective in improving performance and reducing errors in fingerprint comparison as it is in face comparison–despite the generalisable nature of visual comparison [42, 43]. This difference could be due to the different levels of familiarity with face and fingerprint comparison as fingerprints are relatively novel stimuli to novices, but most people have higher familiarity with faces [44, 45]. Regardless, this study provides additional evidence that the low prevalence effect is remarkably difficult to correct as our results add to the list of those that have attempted–but not succeeded–to correct this effect [18, 20, 23–27].

Given the robust nature of the low prevalence effect, it may be unrealistic to attempt to ameliorate this effect in forensic casework. Alternatively, another solution to reducing this effect would be to balance the relative prevalence of matching and non-matching pairs that examiners experience in casework. In some domains, this may be difficult or impossible (e.g., increasing the occurrence of weapons in real-world baggage screening or cancer in mammograms), this approach could be used in forensic science via blind proficiency tests. Most forensic laboratories and organisations require examiners to regularly complete proficiency tests where examiners evaluate ground-truth known samples. Existing proficiency tests have several weaknesses, including being easier than actual casework and their potential for producing experimenter effects as examiners are aware they are being evaluated [46, 47].

Due to these weaknesses, *blind* proficiency tests are strongly advised by numerous scholars, practitioners and agencies–where managers integrate proficiency tests in casework unbeknownst to examiners [48–51]. As this approach could be controlled from an organisational perspective, it would be possible to administer these tests to help ameliorate the low prevalence effect by introducing more non-matching samples into casework flow. This could potentially decrease any response bias examiners may have due to the base-rates of matching and non-matching pairs that examiners experience professionally. This proposal also has empirical support in non-forensic domains: intermittent ‘bursts’ of high-prevalence trials (e.g., a block of trials where the occurrence of weapons is suddenly high) is one of the few approaches that has been shown to ameliorate the low prevalence effect [18, 20, 52]. One additional approach that could reduce this potential source of bias in forensic decision-making is blind verification–emerging research has shown that independent ‘double reading’ can reduce the low prevalence effect in cancer detection in mammograms [53]. Future research should continue to investigate the ways that the low prevalence effect can be mitigated in forensic decision-making–including testing the impact of intermittent ‘bursts’ of high non-match prevalence and verification procedures in visual comparison.

It is important to note that our study did not recruit practising examiners as many studies that recruit forensic professionals do not reach the sample sizes required for experimental work (e.g., *n*s = 11–52; [5, 54–56]; compared to the 116 trainees in the present study). Although we cannot draw explicit conclusions about whether professional forensic examiners are also susceptible to the low prevalence effect, it is important to note that experience and training do not typically ameliorate this potential source of bias. For example, both medical students and fully-qualified doctors are equally susceptible to the low prevalence effect in detecting cancer lesions [28]. Many other studies investigating this effect also use trainee samples–for example, newly-trained TSA baggage screeners [18] or newly-trained security screeners [21]. It is therefore likely that professional forensic examiners are equally susceptible to the low prevalence effect in casework. Nevertheless, it is important that future research replicate this effect in a professional population.

In sum, this study is the first to demonstrate that forensic science trainees are no less susceptible to the low prevalence than novices, and provides further evidence of the robust and difficult-to-correct nature of this effect. Even using a feature-comparison strategy that has been beneficial in other visual comparison domains, both trainees and novices instructed to use this technique were still equally affected by the low prevalence effect. Future work should aim to clarify whether balancing the base-rates of matching and non-matching pairs could ameliorate this potential source of bias in forensic casework, and continue to investigate it in professional forensic examiners.

## Supporting information

S1 Text Supplementary analyses. Supplementary analyses, including table of all statistical results reported in-text, pre-registered analyses utilising our initial pre-registered definition of ‘trainee’, and three additional exploratory analyses investigating novices and trainees with fingerprint training, trainees with and without fingerprint training, and analyses controlling for gender. Note that the results of all three exploratory analyses are consistent with those outlined in-text.
(PDF)
