# Supplementary material for: The low prevalence effect in fingerprint comparison amongst forensic science trainees and novices
Source: PLoS One. 2022 Aug 11;17(8):e0272338. doi: 10.1371/journal.pone.0272338 (PMC9371274; doi:10.1371/journal.pone.0272338)
Supplement: S1 Text — Supplementary analyses, including table of all statistical results reported in-text, pre-registered analyses utilising our initial pre-registered definition of ‘trainee’, and three additional exploratory analyses investigating novices and trainees with fingerprint training, trainees with and without fingerprint training, and analyses controlling for gender. Note that the results of all three exploratory analyses are consistent with those outlined in-text. (PDF) [file pone.0272338.s001.pdf]

Output of All Analyses Reported In-Text

Table 1. Results of exploratory analyses of novices and trainees who reported having training or study in fingerprint examination

False-Alarm Rate Analysis				
	b	*t*	*p*	95% CI
Prevalence	1.71	5.47	< .001	[1.10, 2.59]
Strategy	.38	1.67	.096	[-.07, .83]
Group	-.42	1.84	.065	[-.86, .03]
Prevalence x Strategy	-.58	1.48	.140	[-1.36, .19]
Prevalence x Group	-.01	.03	.980	[-.78, .76]
Strategy x Group	-.26	.84	.402	[-.87, .35]
Prevalence x Strategy x Group	.88	1.57	.117	[-.22, 1.98]
Miss Rate Analysis				
	b	*t*	*p*	
Prevalence	-1.07	5.38	< .001	[-1.45, -.68]
Strategy	-.18	.90	.366	[-.70, .08]
Group	-.31	1.57	.117	[-.57, .21]
Prevalence x Strategy	-.12	.42	.677	[-.67, .43]
Prevalence x Group	-.031	.12	.905	[-.58, .52]
Strategy x Group	.25	.92	.360	[-.28, .78]
Prevalence x Strategy x Group	-.09	.23	.816	[-.87, .69]
Sensitivity Analysis				
	b	*t*	*p*	
Prevalence	-.11	.96	.341	[-.33, .11]
Strategy	-.07	.64	.525	[.15, .58]
Group	.36	3.28	.001	[-.30, .15]
Prevalence x Strategy	.31	1.96	.051	[-.02, .63]
Prevalence x Group	.07	.44	.659	[-.38, .24]
Strategy x Group	-.01	.07	.943	[-.31, .29]
Prevalence x Strategy x Group	-.28	1.24	.218	[-.72, .16]
Response Bias Analysis				
	b	*t*	*p*	
Prevalence	-.64	6.66	< .001	[-.83, -.45]
Strategy	-.15	1.56	.121	[-.34, .04]
Group	.02	.24	.811	[-.17, .21]
Prevalence x Strategy	.11	.77	.440	[-.17, .38]
Prevalence x Group	-.03	.24	.809	[-.30, .24]
Strategy x Group	.13	.99	.322	[-.13, .39]
Prevalence x Strategy x Group	-.21	1.10	.272	[-.59, .17]

Pre-Registered Analyses

We compared error rates, sensitivity and bias between novices (*n* = 114) and forensic science trainees who reported being interested in pursuing a career in fingerprint identification in the future (*n* = 53) as per our pre-registered definition. All trainees who reported they were not interested were excluded from this analysis (*n* = 58).

Analyses of Error Rates

To investigate the impact of prevalence and fingerprints ratings on fingerprint-matching errors (false alarms and misses), we used the *lmer* (Bates et al., 2014) and *lmerTest* (Kuznetsova et al., 2017) packages in R to create logistic mixed-effects models to predict each measure at the trial level from the interaction between prevalence condition (equal or high), training condition (feature-by-feature comparison or control) and group (novices or trainees). Random effects were included for trial and participant which allowed values to vary between stimuli and participants.

False alarms. We identified a low prevalence effect: the false alarm rate was significantly higher in the low prevalence conditions (*M* = .70, *SD* = .46), than the equal prevalence conditions (*M* = .47, *SD* = .50; *b* = 1.59, *z* = 5.08, $p < .001$, 95% CI [.97, 2.20]). However, both groups were equally susceptible to the effect as the false alarm rate did not significantly differ between groups (*b* = -.39, *z* = 1.48, *p* = .140, 95% CI [-.92, .13]), nor did false alarms significantly differ between training conditions (*b* = .30, *z* = 1.39, *p* = .165, 95% CI [-.13, .73]).

The two-way interactions between prevalence and training (*b* = -.54, *z* = 1.39, *p* = .165, 95% CI [-1.29, .22]), prevalence and group (*b* = .01, *z* = .01, *p* = .989, 95% CI [-1.15, 1.17]), and training and group were also not significant (*b* = .-07, *z* = .20, *p* = .845, 95% CI [-.80, .65]), nor

was the three-way interaction between prevalence, training and group (b = .58, z = .77, p = .441, 95% CI [-.89, 2.04]).

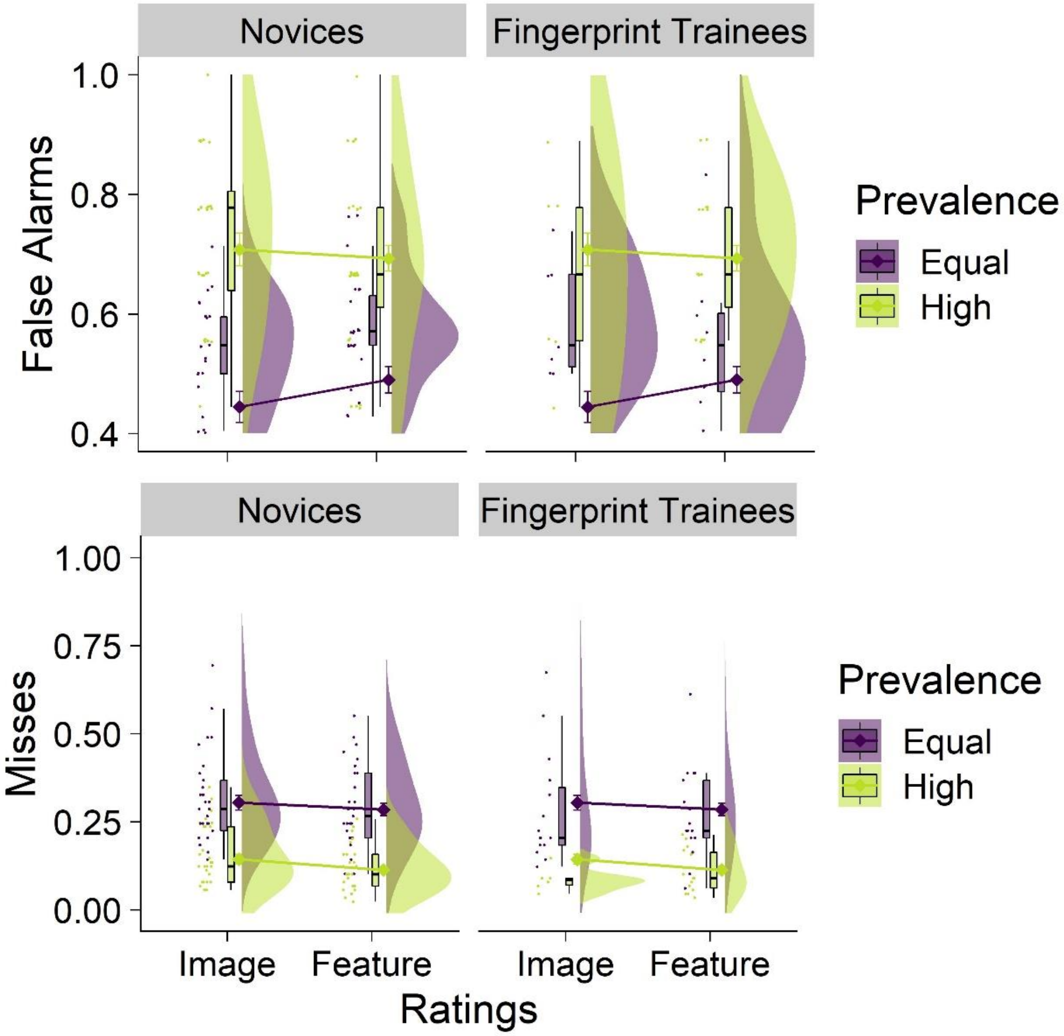

Figure 1. False alarms (top panel) and misses (bottom panel) by prevalence, ratings conditions, and group. Raincloud plots depict (left-to-right) raw jittered data points, box-and-whisker plots, means (represented by diamonds) with error bars representing ± 1 *SE*, and frequency distributions.

Misses. Prevalence also impacted the miss rates as they were significantly lower in the low prevalence conditions (M = .13, SD = .34), than the equal prevalence conditions (M = .30, SD = .46; b = -1.04, z = 5.59, p < .001, 95% CI [-1.40, -.67]). However, the miss rates again did

not significantly differ between groups (*b* = -.23, *z* = 1.01, *p* = .313, 95% CI [-.67, .02]) or training conditions (*b* = -.10, *z* = .52, *p* = .603, 95% CI [-.46, .27]).

The two-way interactions between prevalence and training (*b* = -.24, *z* = .92, *p* = .355, 95% CI [-.76, .27]), prevalence and group (*b* = -.50, *z* = 1.27, *p* = .204, 95% CI [-1.28, .27]), and training and group were also not significant (*b* = .05, *z* = .17, *p* = .862, 95% CI [-.56, .66]), nor was the three-way interaction between prevalence, training, and group was also not significant (*b* = .56, *z* = 1.10, *p* = .271, 95% CI [-.43, 1.54]).

Analyses of Sensitivity and Bias

We also used the *lm* function in the core stats package in R to predict sensitivity and bias from the interaction between the prevalence and fingerprint ratings conditions.

Sensitivity. Sensitivity did not significantly differ between the prevalence (*b* = -.13, *t* = 1.24, *p* = .216, 95% CI [-.35, .08]) or training conditions (*b* = -.08, *t* = .73, *p* = .464, 95% CI [-.29, .13]) – suggesting that neither prevalence or training improved fingerprint comparison performance. However, trainees' sensitivity (*M* = .72, *SD* = .12) was significantly higher than novices (*M* = .70, *SD* = .12; *b* = .31, *t* = 2.38, *p* = .019, 95% CI [.05, .57]) – suggesting that trainees outperformed novices in the fingerprint comparison task.

The two-way interaction between prevalence and training was significant (*b* = .35, *t* = 2.27, *p* = .024, 95% CI [.05, .65]), but none of the follow-up comparisons were not significant. The two-way interactions between prevalence and group (*b* = .13, *t* = .59, *p* = .556, 95% CI [-.31, .58]) and training and group were also not significant (*b* = -.01, *t* = .04, *p* = .965, 95% CI [-.37, .35]), nor was the three-way interaction between prevalence, training, and group (*b* = -.44, *t* = 1.51, *p* = .132, 95% CI [-1.01, .13]).

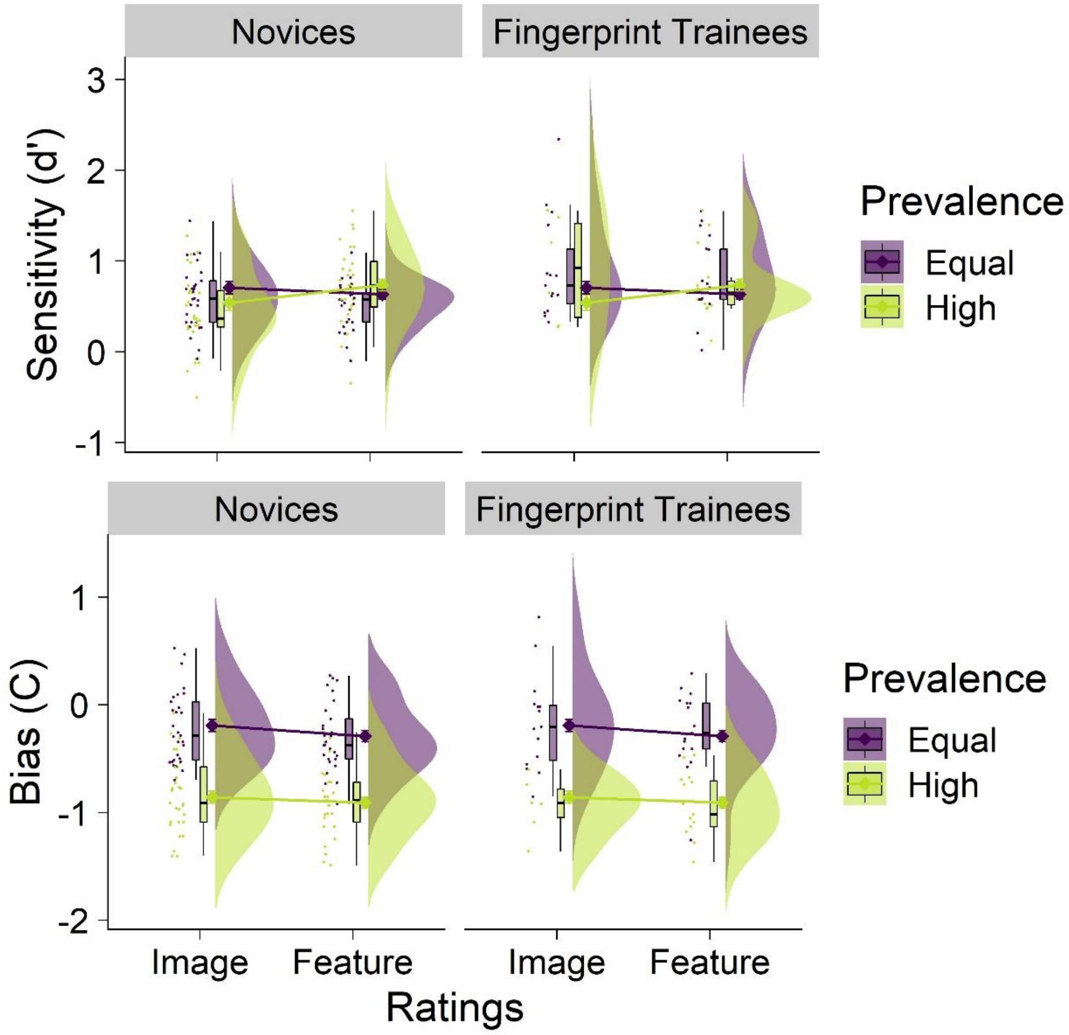

Figure 2. Sensitivity (top panel) and response bias (bottom panel) by prevalence, ratings conditions, and group. Raincloud plots depict (left-to-right) raw jittered data points, box-and-whisker plots, means (represented by diamonds) with error bars representing ± 1 *SE*, and frequency distributions.

Bias.

Prevalence impacted participants' response bias (C) such that it was significantly more liberal in the low prevalence conditions (M = -.89, SD = .32), than equal prevalence conditions (M = -.24, .36; b = -.63, t = 6.98, p < .001, 95% CI [-.81, -.46]. However, both groups were equally susceptible to this shift in response bias as it did not significantly differ between groups (b = .04, t = .39, p = .670, 95% CI [-.17, .26]), nor did training impact response bias as it did not significantly differ between training conditions (b = -.11, t = 1.22, p = .224, 95% CI [-.29, .07]).

The two-way interactions between prevalence and training (b = .06, z = .46, p = .647, 95% CI [-.20, .31]), prevalence and group (b = -.14, t = .73, p = .468, 95% CI [-.51, .24]), and training and group were also not significant (b = .03, t = .19, p = .846, 95% CI [-.27, .33]), nor was the three-way interaction between prevalence, training, and group (b = .02, t = .09, p = .928, 95% CI [-.46, .51]).

Exploratory Analyses

Analyses of Novices and Forensic Trainees with Fingerprint Training

We conducted an exploratory analysis comparing forensic trainees who reported having study or training in fingerprint examination (n = 58), compared to a group of 58 randomly-selected novices. Trainees in this sample were on average 21.18 years old (SD = 2.30, *range* = 18-28), and most (85.96%) self-identified as female (12.28% male; 1.75% gender diverse). The average trainee reported having 23.54 months of experience (SD = 11.59, *range* = 3-45) in their current forensic science degree or certification program. Novices in this sample were on average 36.76 years old (SD = 11.54, *range* = 19-60) and the majority of participants self-identified as female (53.45%; 46.55% male).

To investigate the effects of training, prevalence, and strategy on fingerprint identification errors (false alarms and misses), we conducted create logistic mixed-effects models to predict each measure at the trial level from the interaction between prevalence (equal or high), strategy (feature-comparison or control), and group (novices or trainees), with random effects included for trial and participant. We also conducted linear regression models to predict sensitivity and bias from the interaction between prevalence, strategy, and group. The pattern of results was largely consistent with those reported in the manuscript (see Table 1).

Table 1. Results of exploratory analyses of novices and trainees who reported having training or study in fingerprint examination

False-Alarm Rate Analysis			
	b	*t*	*p*
Prevalence	1.19	3.09	.002
Strategy	.45	1.46	.145
Group	-.37	1.42	.156
Prevalence x Strategy	-.29	.55	.580
Prevalence x Group	.32	.63	.530

Strategy x Group	.30	.72	.474
Prevalence x Strategy x Group	1.47	1.94	.052

Miss Rate Analysis

	b	*t*	*p*
Prevalence	-.93	3.76	< .001
Strategy	-.01	.04	.965
Group	-.23	1.00	.317
Prevalence x Strategy	-.43	1.13	.259
Prevalence x Group	-.32	.86	.389
Strategy x Group	.15	.41	.680
Prevalence x Strategy x Group	.47	.86	.392

Sensitivity Analysis

	b	*t*	*p*
Prevalence	-.03	.25	.806
Strategy	-.20	1.30	.198
Group	.30	2.31	.023
Prevalence x Strategy	.39	.21	.074
Prevalence x Group	.23	.20	.256
Strategy x Group	.06	.30	.768
Prevalence x Strategy x Group	-.84	2.75	.007

Response Bias Analysis

	b	*t*	*p*
Prevalence	-.53	4.60	< .001
Strategy	-.12	.90	.369
Group	-.02	.34	.768
Prevalence x Strategy	-.02	.12	.906
Prevalence x Group	-.02	.11	.906
Strategy x Group	.11	.64	.526
Prevalence x Strategy x Group	-.22	.85	.396

Analyses of Forensic Trainees with and without Fingerprint Training

We conducted an exploratory analysis comparing forensic trainees who reported having study or training in fingerprint examination (n = 58), compared to forensic trainees who reported not having any comparable study or training (n = 58).

Trainees who reported having fingerprint study or training were on average 21.14 years old (*SD* = 2.30, *range* = 18-28), and most (86.21%) self-identified as female (12.07% male;

1.72% gender diverse). These trainees reported having an average of 23.45 months of experience (*SD* = 11.51, *range* = 3-45) in their current forensic science degree or certification program. Trainees who reported not having fingerprint study or training were on average 21.95 years old (*SD* = 5.34, *range* = 18-48), and most (81.03%) self-identified as female (18.97% male). These trainees reported having an average of 16.69 months of experience (*SD* = 11.78, *range* = 2-42) in their current forensic science degree or certification program.

To investigate the effects of training, prevalence, and strategy on fingerprint identification errors (false alarms and misses), we conducted create logistic mixed-effects models to predict each measure at the trial level from the interaction between prevalence (equal or high), strategy (feature-comparison or control), and group (trainees with or without training), with random effects included for trial and participant. We also conducted linear regression models to predict sensitivity and bias from the interaction between prevalence, strategy, and group. The pattern of results was largely consistent with those reported in the manuscript (see Table 2).

Table 2. Results of exploratory analyses of trainees who reported having training or study in fingerprint examination and those who reported no training or study in fingerprint examination

False-Alarm Rate Analysis			
	b	*t*	*p*
Prevalence	1.21	2.73	.006
Strategy	.21	.69	.493
Group	.48	1.32	.186
Prevalence x Strategy	1.13	1.91	.057
Prevalence x Group	1.01	1.67	.095
Strategy x Group	-.38	.81	.417
Prevalence x Strategy x Group	-1.32	1.57	.117

Miss Rate Analysis			
	b	*t*	*p*
Prevalence	1.26	4.29	< .001
Strategy	.14	.53	.596
Group	-.18	.56	.575
Prevalence x Strategy	.04	.09	.929

Prevalence x Group	.34	.77	.443
Strategy x Group	-.03	.08	.933
Prevalence x Strategy x Group	-.60	.99	.320

Sensitivity Analysis

	b	*t*	*p*
Prevalence	.18	1.11	.271
Strategy	-.16	1.14	.259
Group	-.13	.77	.446
Prevalence x Strategy	-.43	1.89	.062
Prevalence x Group	-.55	2.32	.023
Strategy x Group	.20	.89	.373
Prevalence x Strategy x Group	.79	2.43	.017

Response Bias Analysis

	b	*t*	*p*
Prevalence	-.57	3.99	< .001
Strategy	-.02	.17	.866
Group	-.16	1.06	.290
Prevalence x Strategy	-.23	1.11	.270
Prevalence x Group	-.12	.56	.580
Strategy x Group	.08	.40	.690
Prevalence x Strategy x Group	-.12	.41	.681

Analyses of Controlling for Gender

We conducted an exploratory analysis that replicate the results reported in the manuscript but also accounting for gender due to the gender imbalance in the trainee sample. To investigate the effects of training, prevalence, and strategy on fingerprint identification errors (false alarms and misses), we conducted create logistic mixed-effects models to predict each measure at the trial level from the interaction between prevalence (equal or high), strategy (feature-comparison or control), and group (novices or trainees), with random effects included for trial and participant. We also conducted linear regression models to predict sensitivity and bias from the interaction between prevalence, strategy, and group. We included a fixed effect in each analysis to account for gender. The pattern of results was consistent with those reported in the manuscript (see Table 3).

Table 3. Results of exploratory analyses of novices and trainees including gender as a fixed factor in all analyses

False-Alarm Rate Analysis			
	b	*t*	*p*
Prevalence	1.72	5.52	< .001
Strategy	.35	1.52	.129
Group	-.42	1.80	.071
Prevalence x Strategy	-.49	1.23	.220
Prevalence x Group	.01	.04	.971
Strategy x Group	-.18	.57	.567
Prevalence x Strategy x Group	.74	1.32	.186
Miss Rate Analysis			
	b	*t*	*p*
Prevalence	-.1.08	5.46	< .001
Strategy	-.17	.83	.405
Group	-.35	1.72	.085
Prevalence x Strategy	-.17	.59	.553
Prevalence x Group	-.01	.04	.969
Strategy x Group	.23	.84	.401
Prevalence x Strategy x Group	-.04	.11	.915
Sensitivity Analysis			
	b	*t*	*p*
Prevalence	-.11	.97	.333
Strategy	-.06	.51	.611
Group	.34	3.23	.001
Prevalence x Strategy	.30	1.85	.066
Prevalence x Group	-.87	.55	.586
Strategy x Group	-.05	.30	.766
Prevalence x Strategy x Group	-.24	1.07	.286
Response Bias Analysis			
	b	*t*	*p*
Prevalence	-.65	6.78	< .001
Strategy	-.14	1.44	.153
Group	.01	.08	.940
Prevalence x Strategy	.08	.57	.568
Prevalence x Group	-.03	.22	.830
Strategy x Group	.11	.81	.419
Prevalence x Strategy x Group	-.18	.92	.360
